# Sirolimus in combination with low-dose extended-release tacrolimus in kidney transplant recipients

**DOI:** 10.3389/fmed.2023.1281939

**Published:** 2023-12-01

**Authors:** Zhi-yu Zou, Lin-rui Dai, Yi-bo Hou, Chen-zhen Yu, Ren-jie Chen, Yan-yan Chen, Bin Liu, Hui-bo Shi, Nian-qiao Gong, Zhi-shui Chen, Song Chen, Sheng Chang, Wei-jie Zhang

**Affiliations:** ^1^Institute of Organ Transplantation, Tongji Hospital, Tongji Medical College, Huazhong University of Science and Technology, and Key Laboratory of Organ Transplantation, Ministry of Education, and NHC Key Laboratory of Organ Transplantation, and Key Laboratory of Organ Transplantation, Chinese Academy of Medical Sciences, Wuhan, China; ^2^Department of Information Management, Tongji Hospital, Tongji Medical College, Huazhong University of Science and Technology, Wuhan, China

**Keywords:** kidney transplantation, immunosuppressant, medication adherence, sirolimus, tacrolimus

## Abstract

**Introduction:**

Many challenges remain for long-term survival of renal allografts. Once-daily sirolimus (SRL) combined with low-dose extended-release tacrolimus (LER-TAC) may improve medication adherence and reduce the potential nephrotoxicity of calcineurin inhibitors (CNI) compared with standard immunosuppression regimens, thus potentially improving long-term graft survival.

**Methods:**

This retrospective, observational, single-center, propensity score matching (PSM) study compared conversion to SRL combined with low-dose ER-TAC and mycophenolic acid (MPA) combined with standard-dose TAC in kidney transplant recipients. After PSM, there were 56 patients in each group. Efficacy, safety, and medication adherence were evaluated over 12 months.

**Results:**

There was no significant difference between the two groups in terms of graft and recipient survival and incidence of biopsy-proven acute rejection (*p* = 1.000), and none of the recipients developed dnDSA after conversion. The mean eGFR improved in SRL + LER-TAC group after conversion compared to before conversion (51.12 ± 20.1 ml/min/1.73 m^2^ vs. 56.97 ± 19.23 ml/min/1.73 m^2^, *p* < 0.05). The medication adherence at 12 months after conversion was superior to before conversion (*p* = 0.002).

**Discussion:**

Our findings suggest that an immunosuppressive regimen of SRL combined with low-dose ER-TAC is no less effective and safe than standard immunosuppressive regimens for renal transplant recipients and may improve graft renal function and medication adherence.

## 1. Introduction

Kidney transplantation(KT) remains one of the most promising approaches for the treatment of end-stage renal disease (ESRD) (1). Data from the US Organ Procurement and Transplantation Network (OPTN) and the Scientific Registry of Transplant Recipients (SRTR) had shown a 1-year all-cause graft failure rate of less than 10% among deceased donor kidney transplant recipients (KTR), whereas a 10-year all-cause graft failure rate of 40–60% (2–4). Improving graft function and recipient health would not only improve quality and length of life but also reduce the need for retransplantation. However, many challenges remain in improving long-term prognosis.

COMMIT (the Consensus on Managing Modifiable Risk in Transplantation) demonstrated that nonadherence, under immunosuppression, toxicity and adverse effects related to immunosuppression, and high intra-patient variability (IPV) were modifiable risk factors related to immunosuppression for graft failure over the longer term. COMMIT also recommended that simplified drug regimens, such as once-daily dosing, should be administered to improve adherence with Level 1 evidence grade according to the Oxford Centre for Evidence-Based Medicine (OCEBM) system (5).

Calcineurin inhibitors (CNIs), including TAC and cyclosporine (CsA), combined with mycophenolate acid (MPA) and steroids are considered the current standard immunosuppression protocol for kidney transplant recipients, which had significantly reduced the rate of acute rejection and yielded excellent short-to-medium-term graft survival (6). Immediate-release tacrolimus (Prograf®, Astellas Ireland Co. Ltd., IR-TAC) is administered twice daily, whereas extended-release tacrolimus (Advagraf®, Astellas Ireland Co. Ltd., ER-TAC) allows once-daily dosing, which had the potential to improve treatment adherence and reduce significant interactions between TAC and other drugs and diets (7, 8). However, MPA does not allow for once-daily dosing, so the current mainstream immunosuppression regimen of TAC/MPA/Pred still requires twice-daily dosing. In addition, potential CNI nephrotoxicity becomes one of the risks which affect long-term outcomes. Minimizing the dose of CNIs is one way to ameliorate CNI nephrotoxicity (5).

Mammalian rapamycin (mTOR) inhibitors, including sirolimus (SRL), had been used in clinical renal transplantation since 2001, also allowing once-daily dosing. It impaired lymphocyte activation and proliferation by inhibiting mTOR and also prevents chronic allograft nephropathy (CAN) by inhibiting the proliferation of vascular smooth muscle cells with little to no nephrotoxicity (9). It seemed that SRL-containing regimens had advantages over preserving good renal function and improving long-term graft and patient survival, including antiviral and anticancer effects (10).

SRL in combination with low-dose ER-TAC enabled a once-daily immunosuppressive regimen with significantly improved medication adherence compared to CNI in combination with MPA (8). Attenuation of CNI reduced the potential nephrotoxicity of CNI, while SRL had potential antiviral and antitumor effects (11). This study aimed to compare the efficacy and safety of SRL in combination with low-dose ER-TAC (SRL + LER-TAC) versus MPA in combination with standard-dose TAC (MPA + STAC) regimen in kidney transplant recipients.

## 2. Materials and methods

### 2.1. Study design

This retrospective study included patients who received deceased donor kidneys between January 2017 and September 2021. The main inclusion criteria included stable KT recipients, deceased donor KT recipients and *de novo* KT patients. Stable recipients (≥18 years) were defined as kidney transplant recipients who had no acute rejection episodes and no >10% increase in serum creatinine in the past 3 months. Major exclusion criteria included pediatric recipients aged <18 years; multi-organ transplant recipients; living donor KT recipients; patients with preformed donor-specific anti-HLA antibodies (DSA); retransplantation recipients; urinary albumin creatinine ratio (UACR) was >300 mg/g; triglyceride ≥400 mg/dL (≥4.6 mmol/L) and total cholesterol ≥300 mg/dL (≥7.8 mmol/L). A total of 56 KT recipients who converted from an immunosuppressive regimen of MPA + STAC to SRL + LER-TAC in the stable phase were included in the SRL + LER-TAC group. By propensity score matching, we matched 56 patients on the MPA + STAC immunosuppression regimen as the MPA + STAC group from KT recipients followed at our institution. Of these recipients, 22 were converted due to high basal creatinine and 34 were converted to simplify the immunosuppression program and thereby improve their lifestyles. The postoperative months at conversion in the SRL + LER-TAC group were considered as the study starting point. The start months of follow-up in the MPA + STAC group were the same as the postoperative months at conversion of PSM-matched SRL + LER-TAC group recipients as the baseline, and thereafter 12 months of follow-up.

This study was conducted under the Declaration of Helsinki. All subjects provided written informed consent. The study protocol was approved by the Ethics Committee of Tongji Hospital, Tongji Medical College, Huazhong University of Science and Technology (TJ-IRB20220807).

### 2.2. Immunosuppression

All patients who underwent KT surgery were treated with basiliximab or thymoglobulin for immunosuppressive induction therapy. A Standard TAC-based triple immunosuppressive regimen was applied to patients in the MPA + STAC group, including, MPA and steroids. The target TAC trough levels in blood were 7–10 ng/mL for the first 1 year and 6–8 ng/mL thereafter. Measure the TAC trough levels and adjust the dose keeping the trough level within the target range at every outpatient visit. Mycophenolate mofetil (MMF) at 500–750 mg (or enteric-coated mycophenolate sodium at 360–540 mg) was administered orally twice daily. In the SRL+ LER-TAC group, the same immunosuppressive protocol before the conversion was converted to a modified once-daily immunosuppressive regimen including SRL and steroids during routine clinical practice. Twice-daily IR-TAC was converted to once-daily ER-TAC for a total daily dose of 1:1 mg and MPA was converted to once-daily SRL 2 mg as starting dose. The ER-TAC and SRL doses were adjusted to the target trough level of 3–5 ng/mL and the SRL to 5–7 ng/mL, respectively. Prednisone acetate tablets were maintained at 10 mg once daily in both groups.

### 2.3. Assessments

The study outcomes consisted of efficacy profiles, safety profiles, and medication adherence. The efficacy assessments were composed of biopsy-proven acute rejection (BPAR), patient and graft survival, and graft function. Patients with clinical manifestations suggestive of acute rejection underwent biopsy before initiation of steroid pulse treatment. The Banff 2014 was used to grade the biopsy specimens. *De novo* DSA monitoring was performed on renal transplant recipients before and after conversion to a once-daily immunosuppression regimen. Graft function was assessed by estimated glomerular filtration rate (eGFR) calculated using the MDRD. The safety assessments included incidences of adverse events (AEs). Medication adherence was measured by the ITBS score for adherence to the twice-daily regimen at baseline and adherence to the once-daily regimen at 12 months after conversion.

### 2.4. Sample size and statistical analysis

Data are expressed as mean ± standard deviation for normally distributed variables, median (interquartile range) for non-normally distributed variables, and number (proportion) for categorical variables. Continuous variables were analyzed using a t-test or analysis of variance for normally distributed data and Mann–Whitney U-test for non-normally distributed data. Categorical variables were compared using the *χ*^2^ test or Fisher’s exact test. Repeated measures data were analyzed using repeated measures ANOVA. Bonferroni correction was used to reduce type I error because of the multiple comparisons among multiple time points. To overcome bias from different distributions of covariables among patients in the 2 study groups, propensity score matching (PSM) was performed using logistic regression analysis to create propensity scores for both groups. Covariates used for matching were selected *a priori* that were known to be risk factors for mortality or allograft loss based on clinical judgment and previously published literature (12). The following variables were entered into the propensity model: recipient age/sex/BMI, donor age/sex/BMI, transplant years, cause of ERSD, years on dialysis, induction, donor types [donor after circulatory (DCD) and brainstem death (DBD)] and cold ischemia time. We applied a nearest neighbor matching algorithm using a caliper of 0.01 between the SRL + LER-TAC group and the MPA + STAC group. All statistical analyses were performed using the SPSS software version 26.0. A *value of p*<0.05 was considered statistically significant.

## 3. Results

### 3.1. Study characteristics

Based on propensity score matching 1:1, 56 pairs of kidney transplant recipients were combined between the SRL + LER-TAC group and the MPA + STAC group according to recipient age/sex/BMI, donor age/sex/BMI, transplant years, cause of ERSD, years on dialysis, induction, donor types and cold ischemia time (Table 1). The baseline characteristics of each group are shown in Table 2. Two patients in the SRL+ LER-TAC group were switched to other immunosuppressive regimens due to human parvovirus B19 (HPV-B19) infection (*n* = 1) or BK virus infection (*n* = 1). Two patients in the MPA + STAC group were switched to other immunosuppressive regimens due to BK virus infection (*n* = 2).

**Table 1:** Pre-matching and post-matching of propensity score matching (PSM) variables.

Population	Overall (*n* = 828)		*p*-value	Matched (*n* = 112)		*p*-value
	SRL + LER-TAC (*n* = 56)	MPA + STAC (*n* = 772)		SRL + LER-TAC (*n* = 56)	MPA + STAC (*n* = 56)	
*Recipient variables*						
Age (year)	40.5 ± 11.6	41.0 ± 10.7	0.772	40.5 ± 11.6	39.8 ± 10.8	0.310
Male recipient *n* (%)	44 (78.6)	546 (70.7)	0.210	44 (78.6)	44 (78.6)	1.000
BMI (kg/m ^2^)	21.6 ± 3.8	21.6 ± 3.4	0.949	21.6 ± 3.8	21.5 ± 3.3	0.649
Transplant years	2.6 ± 1.3	2.7 ± 1.3	0.521	2.6 ± 1.3	3.0 ± 1.5	0.902
Cause of ERSD *n* (%)			0.528			0.845
Polycystic kidney disease	3 (5.4)	33 (4.3)		3 (5.4)	3 (5.4)	
Nephrolith	3 (5.4)	35 (4.5)		3 (5.4)	4 (7.1)	
Chronic nephritis/nephropathy	21 (37.5)	381 (49.4)		21 (37.5)	26 (46.4)	
Others	1 (5.4)	7 (0.9)		1 (5.4)	1 (5.4)	
Unknown	28(50.0)	316 (40.9)		28 (50.0)	22 (39.3)	
Years on dialysis	2.0 ± 0.8	2.7 ± 1.1	<0.001	2.0 ± 0.8	1.7 ± 0.9	0.508
Induction *n* (%)			0.001			0.699
Basiliximab	35 (62.5)	304 (39.4)		35 (62.5)	33(58.9)	
Thymoglobulin	21 (37.5)	468 (60.6)		21(37.5)	23(41.1)	
*Donor variables*						
Age (year)	47.7 ± 13.9	48.8 ± 11.7	0.491	47.7 ± 13.9	48.6 ± 9.1	0.407
Male recipient *n* (%)	46 (82.1)	634 (82.1)	0.997	46 (82.1)	47 (83.9)	0.801
BMI (kg/m ^2^)	22.7 ± 2.7	23.4 ± 3.0	0.097	22.7 ± 2.7	23.2 ± 1.9	0.215
Donor types *n* (%)			0.379			0.508
DBD	6 (10.7)	116 (15.0)		6 (10.7)	4 (7.1)	
DCD	50 (89.3)	656 (85.0)		50 (89.3)	52 (92.9)	
Cold ischemia time (hours)	10.9 ± 1.7	12.5 ± 2.8	<0.001	10.9 ± 1.7	11.2 ± 2.1	0.919

**Table 2:** Baseline demographics and clinical characteristics.

Population	SRL + LER-TAC (*n* = 56)	MPA + STAC (*n* = 56)	*p*-value
*Recipient variables*			
*Basic disease n (%)*			
Hypertension	50(89.3)	42(75.0)	0.082
Diabetes	2(3.6)	4(7.1)	0.401
Coronary heart disease	2(3.6)	1(1.8)	1.000
HBV positive	10(17.9)	5(8.9)	0.267
*Post-transplant months at baseline*	18.7 ± 22.6	18.7 ± 22.6	0.997
*Induction therapy n (%)*			0.699
Basiliximab	35(62.5)	33(58.9)	
Thymoglobulin	21(37.5)	23(41.1)	
*PRA grade n (%)*			
<10(%)	56(100)	56(100)	1.000
*HLA mismatch*	3.39 ± 0.71	3.16 ± 0.73	0.090

Continuous variables are expressed as the mean ± standard deviation and categorical variables are expressed as numbers (%). The postoperative months at conversion in the SRL + LER-TAC group were considered as the baseline. The start months of follow-up in the MPA + STAC group were the same as the postoperative months at conversion of PSM-matched SRL + LER-TAC group recipients as the baseline, and thereafter 12 months of follow-up.

### 3.2. TAC and SRL exposure

**Figure 1:**
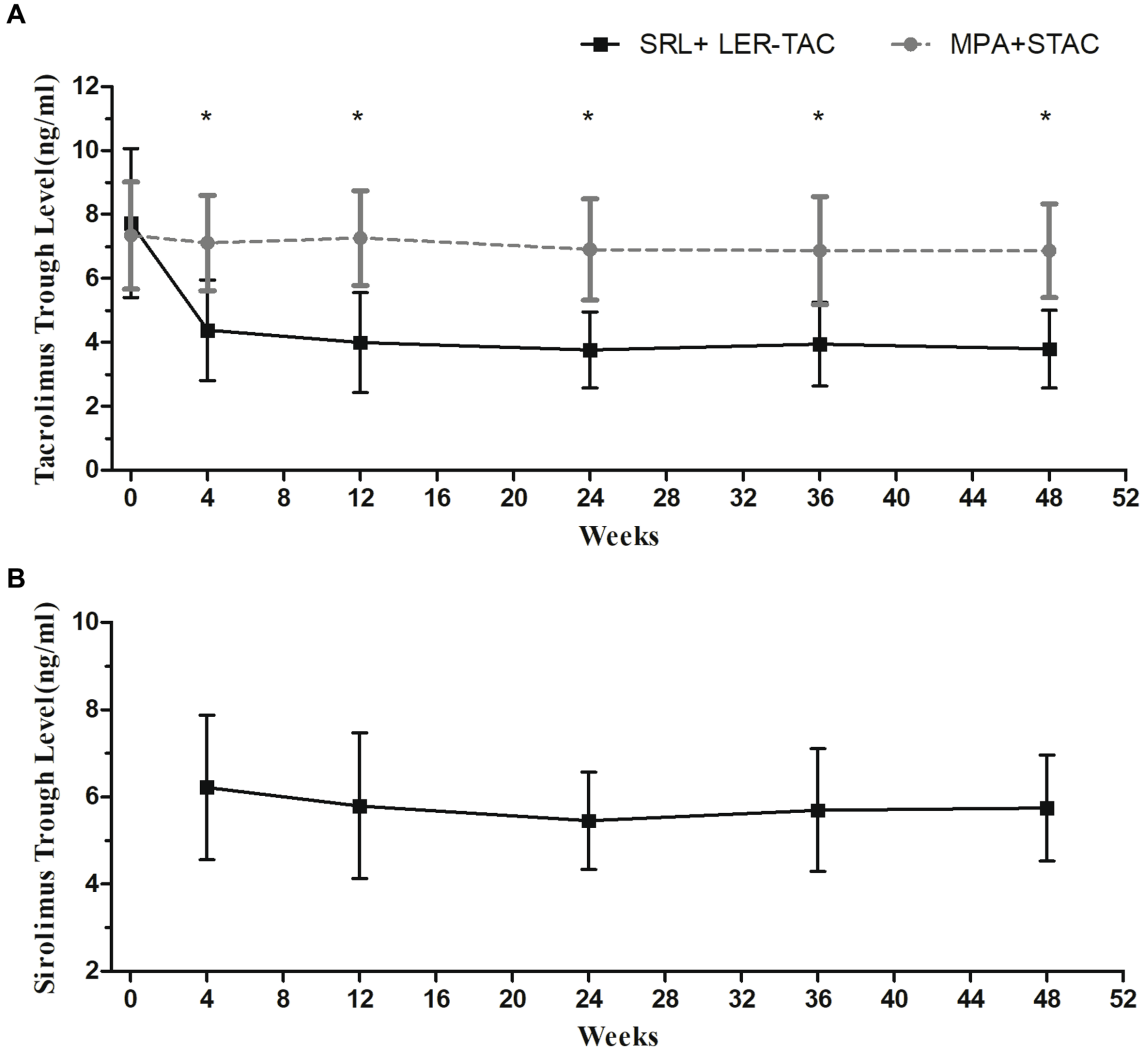
Mean trough levels of TAC and SRL in the two groups. **(A)** TAC trough levels in the SRL + LER-TAC group and MPA + STAC group. **(B)** SRL trough levels in the SRL + LER-TAC group.

The mean trough concentrations of TAC and SRL in both groups were always within the target range. Repeated-measures ANOVA showed that TAC trough concentrations were significantly lower in the SRL + LER-TAC group than in the MPA + STAC groups (*p* < 0.001) and there was a significant time-dependent interaction of the TAC trough concentrations between the two groups (*p* < 0.001). *Post hoc* multiple comparison analysis showed that there was no significant difference in TAC trough concentrations between the two groups at baseline, and there was a significant decrease in TAC trough concentrations in the SRL + LER-TAC group after conversion compared with that before conversion (*p* < 0.001) and a significant difference compared to the MPA + STAC group (*p* < 0.001) (Figure 1).

### 3.3. Graft renal function

**Figure 2:**
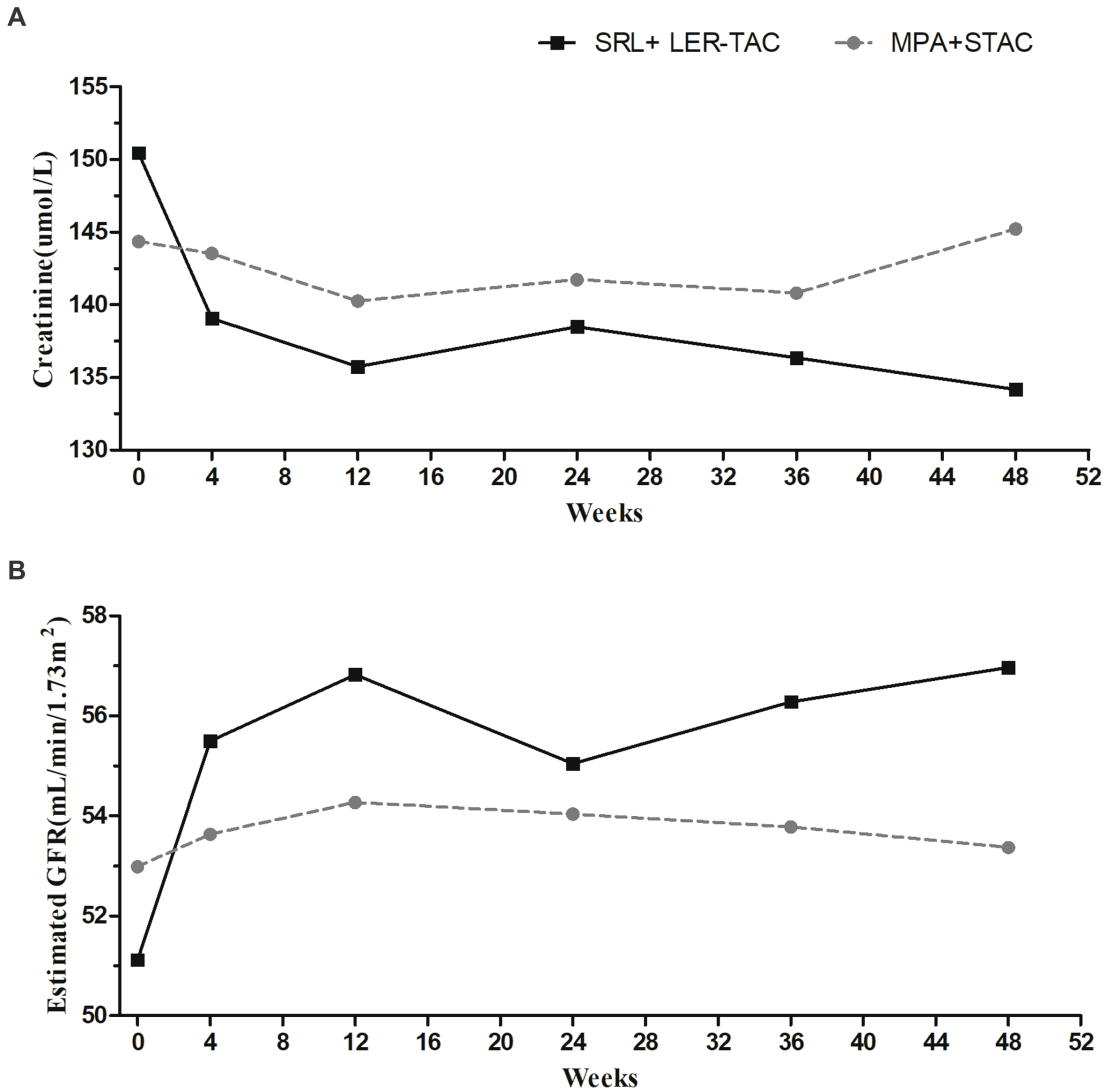
Mean creatinine values and mean estimated glomerular filtration rate in two groups. **(A)** Mean creatinine values in two groups. **(B)** Mean estimated glomerular filtration rate in two groups.

There was no graft loss or patient death in either group, and the graft and patient survival rates were 100% in both groups. Repeated-measures ANOVA showed no significant difference between the mean creatinine values and estimated glomerular filtration rates of the two groups (*F* = 0.230/*p* = 0.632, *F* = 0.270/*p* = 0.605, respectively). However, there were time-dependent interactions of mean creatinine values and estimated glomerular filtration rate between the two groups (*F* = 2.849/*p* = 0.019, *F* = 2.496/*p* = 0.035, respectively). *Post hoc* multiple comparison analysis showed no significant difference in mean creatinine values between the SRL + LER-TAC group and MPA + STAC group from baseline to 12 months of follow-up (baseline: 150.4 ± 47.3 mmoL/L vs. 144.4 ± 39.5 mmoL/L, *F* = 0.545/ *p* = 0.462; 12 months: 134.2 ± 40.0 mmoL/L vs. 145.2 ± 51.8 mmoL/L, *F* = 1.591/*p* = 0.210), but the mean creatinine values in the SRL + LER-TAC group after conversion were significantly lower compared to those before conversion (*F* = 6.037/*p* < 0.001), while there was no significant difference in the mean creatinine values in the MPA + STAC group before and after follow-up (*F* = 1.195/*p* = 0.317). Similarly, from baseline to 12 months of follow-up, there was no significant difference in mean eGFR between the SRL + LER-TAC group and MPA + STAC group (baseline: 51.1 ± 20.1 mL/min/1.73m^2^ vs. 52.9 ± 15.2 mL/min/1.73m^2^, *F* = 0.305/*p* = 0.582; 12 months: 57.0 ± 19.2 mL/min/1.73m^2^ vs. 53.4 ± 15.0 mL/min/1.73m^2^, *F* = 1.219/*p* = 0.272), but the mean eGFR was significantly increased after conversion compared to pre-conversion in the SRL + LER-TAC group (*F* = 6.014/*p* < 0.001), whereas the changes of the mean eGFR during the follow-up period were no significantly different in the MPA + STAC group (*F* = 0.261/*p* = 0.933) (Figure 2).

### 3.4. Biopsy-proven acute rejection and *de novo* DSA

The incidence of BPAR was 3.6% (2/56) in the SRL + LER-TAC group and 1.8% (1/56) in the MPA + STAC group (*p* = 1.000). 2 cases in the SRL + LER-TAC group had BPAR at 2 months and 3 months after conversion, respectively, and 1 case in the MPA + STAC group occurred at 5 months of follow-up. All the 3 cases were TCMR, and the renal allograft function was improved after steroid pulse therapy without complication. In addition, to investigate whether the SRL + LER-TAC regimen increases the risk of *de novo* DSA, we tested all recipients for dnDSA at 3, 6, or 12 months after conversion, including 3 patients who developed TCMR. The results showed none of the recipients developed dnDSA.

### 3.5. Safety assessments

During the study period, the incidence of AE was 76.8% (43/56) in the SRL + LER-TAC group and 87.5% (49/56) in the MPA + STAC group (*p* > 0.05) (Table 3). The most frequently reported AEs included infection (23.2% in the SRL + LER-TAC group and 37.5% in the MPA + STAC group, *p* = 0.100), elevation of liver enzymes (28.6% in the SRL + LER-TAC group and 39.3% in the MPA + STAC group, *p* = 0.231), hyperlipidemia (57.1% in the SRL + LER-TAC group and 44.6% in the MPA + STAC group, *p* = 0.186), glucose intolerance (17.9% in the SRL + LER-TAC group and 23.2% in the MPA + STAC group, *p* = 0.483), proteinuria (48.2% in the SRL + LER-TAC group and 32.1% in the MPA + STAC group, *p* = 0.083). The hyperlipidemia and proteinuria rates were higher in the SRL + LER-TAC group than in the MPA + STAC group, although the difference was not statistically significant (*p* = 0.483, *p* = 0.083, respectively). Urine protein in the SRL + LER-TAC group was all microproteinuria, and the urinary albumin creatinine ratio (UACR) was <300 mg/g. Laboratory values were compared between the two groups at baseline, at 6, and 12 months of follow-up (Table 4). Repeated measures ANOVA showed that there were no significant differences (*p* > 0.05) between the two groups for each laboratory values. The effects of time factor on hemoglobin, cholesterol, UACR were statistically significant (*F* = 23.919/*p* < 0.001, *F* = 9.448/*p* < 0.001, *F* = 3.105/*p* = 0.047, respectively). There were time-dependent interactions of hemoglobin, cholesterol (*F* = 4.218/*p* = 0.017, *F* = 7.569/*p* = 0.001, respectively). *Post hoc* multiple comparison analysis showed that cholesterol levels were statistically higher in the SRL + LER-TAC group than in the MPA + STAC group at 6 months post-conversion (*F* = 4.699/*p* = 0.033).

**Table 3:** Adverse events during the follow-up period.

Adverse events	SRL + LER-TAC (*n* = 56)	MPA + STAC (*n* = 56)	*p*-value
No. of patients with any AE	43 (76.8)	49 (87.5)	0.139
*Infections and infestations*	13 (23.2)	21 (37.5)	0.100
Pulmonary infection	4 (7.1)	8 (14.3)	0.359
Urinary tract infection	10 (17.9)	15 (26.8)	0.257
BKV infection	2 (3.6)	2 (3.6)	1.000
B19 infection	2 (3.6)	0 (0)	0.476
*Metabolism and nutrition disorders*			
Elevation of liver enzymes	16 (28.6)	22 (39.3)	0.231
Hyperlipidemia	32 (57.1)	25 (44.6)	0.186
Glucose intolerance	10 (17.9)	13 (23.2)	0.483
Proteinuria	27 (48.2)	18 (32.1)	0.083
*Hematological disorders*			
Anemia	3 (5.4)	8 (14.3)	0.112
Leukopenia	2 (3.6)	7 (12.5)	0.164
Thrombocytopenia	1 (1.8)	2 (3.6)	1.000

**Table 4:** Laboratory values at baseline, at 6, and 12 months of follow-up.

Laboratory values	SRL + LER-TAC (*n* = 56)	MPA + STAC (*n* = 56)	F_group_/*p*-value	F_time_/*p*-value	F_time*group_/*p*-value
WBC (x10^9^ /l)			1.679/0.198	0.882/0.417	0.206/0.814
Baseline	7.0 ± 2.7	7.3 ± 2.8			
Month 6	6.9 ± 1.7	7.5 ± 2.2			
Month 12	7.2 ± 1.8	7.6 ± 2.7			
Hemoglobin (g/l)			0.305/0.582	23.919/<0.001	4.218/0.017
Baseline	118.1 ± 23.8	125.6 ± 19.7			
Month 6	132.4 ± 20.6	134.1 ± 19.4			
Month 12	137.5 ± 20.3	133.9 ± 19.5			
PLT (x10^9^ /l)			1.216/0.272	2.703/0.072	0.025/0.975
Baseline	222.9 ± 80.9	208.7 ± 65.4			
Month 6	222.3 ± 87.7	209.5 ± 62.0			
Month 12	215.4 ± 77.5	200.8 ± 54.7			
ALT(u/l)			0.047/0.829	1.417/0.247	0.765/0.468
Baseline	22.8 ± 26.4	26.9 ± 20.3			
Month 6	21.8 ± 11.7	20.3 ± 12.0			
Month 12	21.8 ± 11.6	20.6 ± 16.0			
AST(u/l)			0.879/0.350	0.020/0.980	1.407/0.249
Baseline	20.8 ± 17.2	22.4 ± 8.7			
Month 6	22.9 ± 11.6	20.1 ± 6.0			
Month 12	23.2 ± 11.1	20.1 ± 6.5			
Glucose(mmol/l)			1.697/0.195	2.339/0.101	0.564/0.571
Baseline	5.5 ± 0.8	5.8 ± 1.8			
Month 6	5.5 ± 0.7	5.7 ± 1.4			
Month 12	5.4 ± 0.7	5.6 ± 1.2			
Cholesterol (mmol/l)			0.474/0.492	9.448/<0.001	7.569/0.001
Baseline	4.2 ± 1.1	4.5 ± 0.8			
Month 6	4.9 ± 0.9	4.5 ± 0.8			
Month 12	4.7 ± 0.8	4.5 ± 1.0			
Triglyceride (mmol/l)			3.808/0.054	2.884/0.060	1.123/0.329
Baseline	1.6 ± 0.7	1.4 ± 1.0			
Month 6	1.8 ± 0.8	1.5 ± 0.8			
Month 12	1.7 ± 0.8	1.4 ± 0.7			
UACR (mg/g)			0.158/0.692	3.105/0.047	1.221/0.297
Baseline	88.5 ± 142.4	85.8 ± 120.9			
Month 6	107.1 ± 186.9	97.8 ± 160.7			
Month 12	76.2 ± 130.5	80.4 ± 151.3			

The postoperative months at conversion in the SRL + LER-TAC group were considered as the baseline. The start months of follow-up in the MPA + STAC group were the same as the postoperative months at conversion of PSM-matched SRL + LER-TAC group recipients as the baseline, and thereafter 12 months of follow-up.

### 3.6. Medication adherence

Table 5 presents the results of ITBS scores before and 12 months after conversion in the SRL + LER-TAC group. The median total ITBS score before conversion was 16 (12–25) and 12 months after conversion was 14 (12–19) (*p* = 0.043), especially the ITBS scores for the two questions related to frequency of medication administration were significantly lower post-conversion than pre-conversion (*p* < 0.05). Of the 13 questions, “Q1: I have to take the immunosuppressant medication(s) too many times per day “had a median score of 2 (IQR 1–3) before conversion and 1 (IQR 1–2) after conversion (*p* = 0.002); “Q2: I have to take too many capsules (or tablets) of my immunosuppressant medication(s) at one time “had a median pre-conversion score of 2 (IQR 1–3) and a median post-conversion score of 1 (IQR 1–2), (*p* = 0.023). A decrease in scores after conversion to the simplified once-daily regimen was demonstrated by the total ITBS score and 2 ITBS subscales including the “uncontrollable” factor, suggesting that the simplified once-daily regimen may facilitate the reduction of barriers to medication adherence and improve medication adherence in kidney transplant recipients.

**Table 5:** Results of ITBS (Immunosuppressant Therapy Barrier Scale^a^) score.

ITBS Questions	Before	12 months after	*p*-value
“Uncontrollable” factor			
1. I have to take the immunosuppressant medication(s) too many times per day.	2 (1–3)	1 (1–2)	0.002
2. I have to take too many capsules (or tablets) of my immunosuppressant medication(s) at one time.	2 (1–3)	1 (1–2)	0.023
3. I cannot tell if my immunosuppressant medication(s) is (are) helping me.	1 (1–2)	1 (1–2)	0.455
4. I skip doses of my immunosuppressant medication(s) when I go out of town.	1 (1–2)	1 (1–2)	0.560
5. I miss doses of my immunosuppressant medication(s) when I feel depressed.	1 (1–2)	1 (1–2)	0.567
6. I get confused about how to take my immunosuppressant medication.	1 (1–2)	1 (1–1)	0.304
7. I do not understand when to take my immunosuppressant medication(s).	1 (1–2)	1 (1–1)	0.254
8. I often run out (or do not have enough) of immunosuppressant medication(s).	1 (1–2)	1 (1–1)	0.235
“Controllable” factor			
9. It is hard for me to remember to take my immunosuppressant medication(s).	1 (1–2)	1 (1–1)	0.230
10. I miss a dose of my immunosuppressant medication(s) when I think there may be side effects	1 (1–2)	1 (1–2)	0.317
11. I sometimes skip doses of my immunosuppressant medication(s) when I feel good (or better)	1 (1–2)	1 (1–1)	0.317
12. I miss doses of my immunosuppressant medication(s) when I get out of my daily routine	1 (1–2)	1 (1–1)	0.154
13. I skip doses of my immunosuppressant medication(s) when I am short of money	1 (1–2)	1 (1–1)	0.338
Total	16 (13–26)	14 (13–20)	0.043

a Scale grades: 1 ‘strongly disagree’; 2 ‘disagree’; 3 ‘neutral’; 4 ‘agree’; 5 ‘strongly agree’. The scores range from 13 to 65, with a higher score corresponding to more barriers to adherence.

## 4. Discussion

In this study, we presented the results of our retrospective propensity score-matched cohort analysis comparing safety, efficacy, and medication adherence of low-dose ER-TAC combined with SRL (LER-TAC + SRL) versus standard-dose TAC combined with MPA (STAC+ MPA) in stable kidney transplant recipients. Our study suggested that low-dose ER-TAC combined with SRL was non-inferior to standard-dose TAC combined with MPA in terms of efficacy and safety when used as the immunosuppressant regime in patients undergoing kidney transplantation. The lower total ITBS score at 1-year post-conversion compared to pre-conversion suggests that conversion to this simplified once-daily immunosuppressive regimen may improve patient medication adherence.

Graft renal function was stable in both groups throughout the study. Mean eGFR at month 12 of follow-up was 56.97 ± 19.23 mL/min/1.73m^2^ in the SRL + LER-TAC group and 53.37 ± 15.00 mL/min/1.73m^2^ in the MPA + STAC group(*p* = 0.272), similar to that seen in the MPA + TAC group of the SYMPHONY study (54 mL/min/ 1.73 m^2^ by MDRD formula) (13). Although there was no statistically significant difference in eGFR values between the two groups, we found that the mean estimated glomerular filtration rate was significantly increased after conversion compared to pre-conversion in the SRL + LER-TAC group (*p* < 0.05). *P*ost hoc analysis of results from the large randomized A2309 trial showed that the renal dysfunction was predominantly driven by increased exposure to TAC, with inferior renal outcomes above 4 ng/mL and a further increased risk above 6 ng/mL, at month 12 after kidney transplantation in everolimus (EVL)-treated individuals (14). One report on the evolution of SRL-based immunosuppression strategies noted that SRL-containing regimens were associated with the maintenance of good renal function and had promising characteristics for improving long-term survival of both grafts and patients, including antiviral and anticancer effects. It also considered that the use of low-dose SRL (target C_0_ 4–6 ng/mL) in combination with TAC (target C_0_ 3–5 ng/mL)/steroid was an acceptable optimal immunosuppressive strategy (10). For all patients converted to SRL + LER-TAC in this study, the goal of maintaining SRL whole blood trough levels between 5 and 7 ng/mL allowed us to adjust the TAC trough concentration to 3–5 ng/mL, significantly lower than in the MPA + STAC group. Graft renal function in the SRL + LER-TAC group improved after conversion compared with pre-conversion, possibly related to CNI reduction. Although the difference in renal function between the two groups was not statistically significant at 12-month follow-up, further expansion of the sample size and prolongation of follow-up may yield statistically significant results.

In the present study, the difference in the incidence of BPAR between the two groups did not reach statistical significance. The results showed none of the recipients (including 3 BPAR cases) developed dnDSA at 3, 6, or 12 months of follow-up. According to our results, SRL combined with low-dose ER-TAC could be an effective immunosuppressive strategy to prevent acute rejection in kidney transplant recipients which was consistent with the results of the TRANSFORM test (15–17). Similarly, other studies reported non-inferior efficacy and safety of reduced-exposure ER-TAC in combination with mTOR inhibitors versus TAC in combination with MPA (9, 10, 14, 18–21). One study supported maintaining an EVL trough concentration of 3–8 ng/mL combined with low-dose TAC, to achieve balanced efficacy and safety in renal transplant recipients (14). It was demonstrated in trials such as US92, TRANSFORM, and ATHENA that there was no difference in hard outcomes when EVL was used at optimal trough levels (3–8 ng/mL) in combination with reduced doses of CNI (6, 15–17, 22). When SRL was used instead of EVL, as in the RECORD trial, the results are also comparable (9). Subsequent research had shown that a once-daily immunosuppressive regimen of SRL in combination with low-dose ER-TAC can not only effectively prevent acute rejection and preserve renal function but also significantly improve medication adherence (8–10). However, several previous reports had shown that TAC in combination with SRL was associated with worse post-transplant outcomes in terms of patient and graft survival, BPAR, and graft renal function compared to TAC in combination with MPA. This may be related to the lack of early experience with mTORi and synergistic nephrotoxicity and side effects of the combination of inadequate early high-dosing regimens, which may contributed to these disappointing results in terms of renal function and graft survival (23–28). In fact, when SRL was used at optimal trough levels (3–8 ng/mL) in combination with low-dose CNI, there was no difference in terms of hard outcomes and renal function (6, 27, 29).

The safety findings were similar between the two groups in this study, which was consistent with the known safety profiles (9, 21, 22, 30). Several reports had shown that mTOR inhibitors with low-dose CNI reduce the incidence of infection, particularly the risk of viral infections including CMV and BKV (10, 31, 32). Our results also found a lower infection rate in the SRL + LER-TAC group compared to the MPA + STAC group, but no significant differences were seen between the two groups.

The reported side effects of SRL producing abnormal lipid metabolism and proteinuria were relatively clear, and the side effects caused by SRL may be dose-related (33, 34). In this study, we observed that the prevalence of proteinuria and abnormal lipid metabolism was higher in the SRL + LER-TAC group than in the MPA + STAC group, although the difference was not statistically significant. In the SRL + LER-TAC group, an increase in lipid level was observed 6 months after conversion, and after diet and exercise control and lipid-lowering drug treatment, the lipid level could be gradually stabilized, and the lipid level 12 months after conversion was normal. Although hyperlipidemia was manageable in this study, whether it could exacerbate the effects on cardiovascular disease is still unknown and needs to be further explored. However, some studies had confirmed that SRL could delay cardiovascular disease progression (35). The same trend could be found in UACR, proteinuria in the SRL+ LER-TAC group was all microproteinuria, and proteinuria abnormalities were mostly controlled after receiving urinary protein reduction therapy at 12 months after conversion. A *post hoc* analysis demonstrated that the patients who benefited most from conversion were those with a baseline GFR > 40 mL/min and a urine protein to creatinine ratio ≤ 0.11 (36).

Medication adherence for renal transplant recipients in the SRL + LER-TAC group was assessed by ITBS in this study at pre-conversion and 1-year post-conversion, respectively. The results of the study showed a lower total ITBS score at 1-year post-conversion compared to pre-conversion, suggesting that conversion to this simplified once-daily immunosuppressive regimen may improve patient medication adherence. Previous studies had shown that reducing medication frequency improves medication adherence (37), and it was also confirmed in a study of adherence with a simplified once-daily immunosuppressive regimen (8). Our present results were consistent with the previous ones. It suggested that the SRL + LER-TAC regimen may improve patient medication adherence and patient satisfaction, which may improve long-term outcomes of renal transplantation.

Limitations of this study should be noted. Firstly, the present study was retrospective observational, and all renal transplant recipients in the SRL + LER-TAC group were converted during the stable phase rather than the starting application, and the postoperative months at the time of conversion were inconsistent. Although we used propensity score matching in order to minimize bias, unmeasured confounding factors may have influenced our results. Therefore, prospective randomized multicenter studies are necessary to further clarify the efficacy and safety of applying SRL + LER-TAC immunization regimen at initiation or conversion in the stable phase. Secondly, there were limitations in terms of sample size and follow-up time, future studies are necessary to increase the sample size, and should discuss the number of patients required for safe implementation in the clinic based on statistical estimation and assess long-term efficacy and safety as well as adherence. In contrast to Europe and the United States, the major causes of end-stage renal disease in China were predominantly referred to as chronic glomerulonephritis and were predominantly those with a BMI < 28 kg/m^2^. Whereas in Europe and the United States, high-weight obese people with end-stage renal disease combined with metabolic diseases such as diabetes and hyperlipidemia accounted for significantly more of the population requiring kidney transplantation than in China. Therefore, in the application in white populations in Europe and the United States, factors such as diabetes, hyperlipidemia, or obesity amplified the adverse effects of the SRL combined with a low-dose ER-TAC regimen. However, this situation was not significant in this study. Therefore, SRL combined with a reduced dose of ER-TAC may be an ideal protocol for Chinese.

In conclusion, our findings suggest that an immunosuppressive regimen of SRL combined with low-dose ER-TAC is no less effective and safe than standard immunosuppressive regimens for renal transplant recipients. In addition, the conversion regimen also has a significant effect on the improvement of renal function. Furthermore, SRL combined with low-dose ER-TAC allows for once-daily dosing, which may improve patient adherence and have a favorable impact on the long-term prognosis of patients.

## Data availability statement

The original contributions presented in the study are included in the article/supplementary material, further inquiries can be directed to the corresponding authors.

## Ethics statement

The studies involving humans were approved by the Ethics Committee of Tongji Hospital, Tongji Medical College, Huazhong University of Science and Technology (TJ-IRB20220807). The studies were conducted in accordance with the local legislation and institutional requirements. The participants provided their written informed consent to participate in this study.

## Author contributions

Z-yZ: Investigation, Writing – original draft, Writing – review & editing, Methodology, Project administration. L-rD: Data curation, Investigation, Writing – review & editing. Y-bH: Data curation, Investigation, Writing – review & editing. C-zY: Investigation, Writing – review & editing. R-jC: Investigation, Writing – review & editing. Y-yC: Funding acquisition, Resources, Software, Writing – review & editing. BL: Data curation, Writing – review & editing. H-bS: Data curation, Writing – review & editing. N-qG: Data curation, Writing – review & editing. Z-sC: Data curation, Writing – review & editing. SoC: Supervision, Validation, Writing – review & editing. ShC: Methodology, Project administration, Supervision, Validation, Writing – review & editing. W-jZ: Methodology, Project administration, Supervision, Validation, Visualization, Writing – review & editing.
